# Ingenol mebutate treatment in keloids

**DOI:** 10.1186/s13104-015-1429-9

**Published:** 2015-09-22

**Authors:** Bruna De Felice, Marco Guida, Luigi Boccia, Massimo Nacca

**Affiliations:** DISTABIF–Department of Environmental, Biological and Pharmaceutical Sciences and Technologies, University of Naples II, Via Vivaldi 43, 81100 Caserta, Italy; Faculty of Biology, Federico II University of Naples, Naples, Italy; A.O.R.N. Sant’Anna e San Sebastiano Caserta, 81100 Caserta, Italy

**Keywords:** Keloids, p53. ΔΝp63, Ingenol-mebutate

## Abstract

**Background:**

Ingenol-mebutate has been used for the treatment of actinic keratosis. It has been shown that ingenol-mebutate inhibits the growth of cancer cells or induces tumor cell death through pro-apoptotic effects. Keloids are benign skin tumours and are the effect of a deregulated wound-healing process in genetically predisposed patients. Increased cell proliferation, which accounts for the progressive and hypertrophic nature of keloids, correlates with the failure of apoptosis and plays a role in the process of pathological scarring. Keloid cells show a mutated p53 gene resulting in functionally inactive p53 protein which cannot control genomic integrity. They tend to escape from apoptosis which leads to keloid development by means of accumulation of continuously proliferating cells. Currently, the treatment of keloids remains a challenge for high recurrence rates. However, the design and the development of pro-apoptotic therapeutic strategies would be beneficial to keloids treatment.

**Case presentation:**

A 55-year-old caucasian woman presented recurrent keloids on a presternal scar. Standard surgical intervention was used to treat the scar. However, this was unsuccessful and a year later the patient sought treatment again, but only by alternative means as the patient refused further surgical intervention. Consequently, based on past research and experience, the authors attempted to treat these lesions with ingenol mebutate gel, due to the pro-apoptotic effects.

**Conclusion:**

After 1 month, there was a clinical resolution of lesions, with a slightly squamous, post-inflammatory erythema. A cutaneous biopsy proved the absence of residual keloids and deregulated expression of molecular markers. The last follow-up of the patient, 1 year after treatment, showed that the patient was still free of keloids recurrence.

**Electronic supplementary material:**

The online version of this article (doi:10.1186/s13104-015-1429-9) contains supplementary material, which is available to authorized users.

## Background

Ingenol-mebutate has been used in recent treatments of actinic keratosis (also called solar keratosis or dyskeratosis). Ingenol mebutate is a diterpene ester, hydrophobic, isolated from the plant Euphorbia peplum, which at concentrations higher than 200 μmol/L, diffuses through the skin within a few hours after application, concentrating mainly in the epidermis and into the dermis to a lesser extent. The exact mechanism of action is unknown. It is believed that the active ingredient has pleiotropic effects inhibiting the growth of cancer cells or induces tumor cell death through multiple mechanisms. ‘In vivo and in vitro’ models have shown a dual mechanism of action [1]: direct cell death is quickly expressed in tumoral keratinocytes and performs its function with the entry of the molecule into the cell by lysis of pinosomi. Studies with immuno-fluorescence have shown, within the cell, in response to ingenol-3-angelate, PKC-δ (protein kinase C-delta) translocation from the cytosol to the peri-nuclear membrane, [2]. The disruption of the plasma membrane, as demonstrated by electron microscopy, leads to an increase in the intracellular concentration of calcium ions, which, for the gradient, penetrate into the mitochondria causing mitochondrial swelling and lysis [2]. The lack of production of ATP, following the destruction of mitochondria, determines cell necrosis. Beside the pro-apoptotic effects, immuno-stimulant effects are documented due to an increased release of chemokines by activated T cells, local infiltration of neutrophils with increased antibody-dependent cytotoxicity, and finally, the development of specific autoimmune responses of CD8 (+) T cells. The inflammatory reaction determined in the dermis, induces an activation and infiltration of immuno-competent cells as neutrophils, with primary necrosis of tumor cells. It has been demonstrated, in fact, that the exposure of human keratinocytes in vitro to ingenol mebutate, causes the release of chemokines and pro-inflammatory cytokine TNF-α (Tumor necrosis factor-alpha), leading to neutrophil activation mediated by PKC [2].

Direct pro-apoptotic effects of this drug have been demonstrated in several malignant cells, including melanoma cell lines [3]. Topical application of ingenol mebutate was revealed as being effective for human and murine melanoma in mouse models. Further preclinical murine modeling has demonstrated that, through P-glycoprotein-mediated absorptive drug transport, ingenol mebutate can pass the stratum corneum barrier and penetrate to exert its pharmacological effects in the dermis and hypodermis [4]. Ingenol mebutate treatment also reduced the number of mutant p53 keratinocyte patches by about 70 %. No published data are available on a potential effect of ingenol mebutate on keloids [5].

Keloids are benign skin tumours and are the effect of a deregulated wound-healing process in genetically predisposed patients. They are characterized by formation of excess scar tissue beyond the boundaries of the wound. Keloids are often confused with hypertrophic scars because of an apparent lack of morphologic differences.

We already found the extent of apoptosis by DNA fragmentation and MTT assays, propidium iodide staining, p53 expression, and subcellular distribution [6–9]. We also found that selenocystine, a nutritionally available seleno-amino acid, was identified for the first time as a novel agent with anti proliferative activity on human keloids. The 20 μM concentration after 48 h treatment used here was the most effective to reduce keloid fibroblast growth [10].

In a previous research [8], we addressed the mechanisms by which keloids, in contrast to hypertrophic scars, escape from apoptosis. Our data demonstrated that p53 under-expression, due to the sequence mutations, in concert with ΔNp63 activation is the central mechanism involved in keloid proliferation and explains the aberrant growth. ΔNp63 works as a dominant-negative protein upon p53 preventing the latter from activating its target genes in apoptosis process suppression of apoptosis contribute to keloid development by means of accumulation of continuously proliferating cells whereas the disruption or elimination of genetically altered cells might decrease tumor potential. We also showed that p53 and ΔΝp63 deregulated expression can present prognostic relevance as differential apoptosis markers in human keloids.

Here, we describe a case report of a woman with a large keloid on a presternal scar (Additional file 1). The original treatment was unsuccessful the patient categorically refused further surgical intervention. Consequently, based on past research, we decided the best option was to treat such keloid lesions, for the first time, with ingenol mebutate gel. We also assessed ΔΝp63 and p53 altered expression in a cutaneous biopsy from the patient before and 1 month after treatment.

## Consent

Written informed consent was obtained from the patient for publication of this case report and any accompanying images. A copy of the written consent is available for review by the Editor-in-Chief of this journal

We obtained ethics approval for our study from the ethics committee of the University of Naples II, Italy. All procedures used in the study were in accordance with the current international guidelines, with the standards on human experimentation of the Ethics Committee of the University of Naples II, Italy, and with the Helsinki Declaration of 1975, revised in 1983.

## Case presentation

### Initial presentation and diagnostics

A 55-year-old caucasian woman presented with biopsy proven recurrent keloids on a presternal scar, subsequent to intervention of sternotomy, located from manubrio-sternal joint to xiphoid appendix.

The patient had been subjected to sternotomy to treat a severe form of heart valve insufficiency in the year before the treatment. The wounds caused by this surgery, for no apparent underlying cause, evolved spontaneously in a tissue hypertrophy with a violent inflammation and with symptoms of itching and burning. Within 2 months the scar markedly increased, overflowing from the edges of the wound.

As the patient refused to undergo further surgery, we carried out three intralesional fortnightly injections of triamcinolone acetonide 40 mg/ml, which led initial atrophy and telangiectasia, without reducing the symptoms.

Before starting treatment, the clinical appearance was confirmed by taking a skin biopsy punch with 4 mm to presternal region. The diagnosis of a keloid was rather simple and consisted of a simple examination of the lesion and a histological examination, which confirmed the diagnosis of keloid. We also assessed ΔΝp63 and p53 altered expression which confirmed the diagnosis of keloids (data not shown).

## Treatment

Since the previous treatment was unsuccessful and the patient refused any further surgical intervention, the authors decided to attempt to treat the lesions with 500 µg/g gel of ingenol mebutate (everyday applications for 2 days), based on in vitro data on keloids cell lines [5–9].

The exact mechanism of action of ingenol mebutate is not clear yet, but it has proven to be effective in removing actinic keratosis. After the application of ingenol mebutate, the majority of patients (over 95 %) reported one or more skin reactions at the local level such as itching and burning caused by a large inflammation, which is resolved within 30 days with a frequent resolution of the underlying disease. Treatment with ingenol mebutate has no contraindications except hypersensitivity to the active substance or to any of the excipients.

**Fig. 1:**
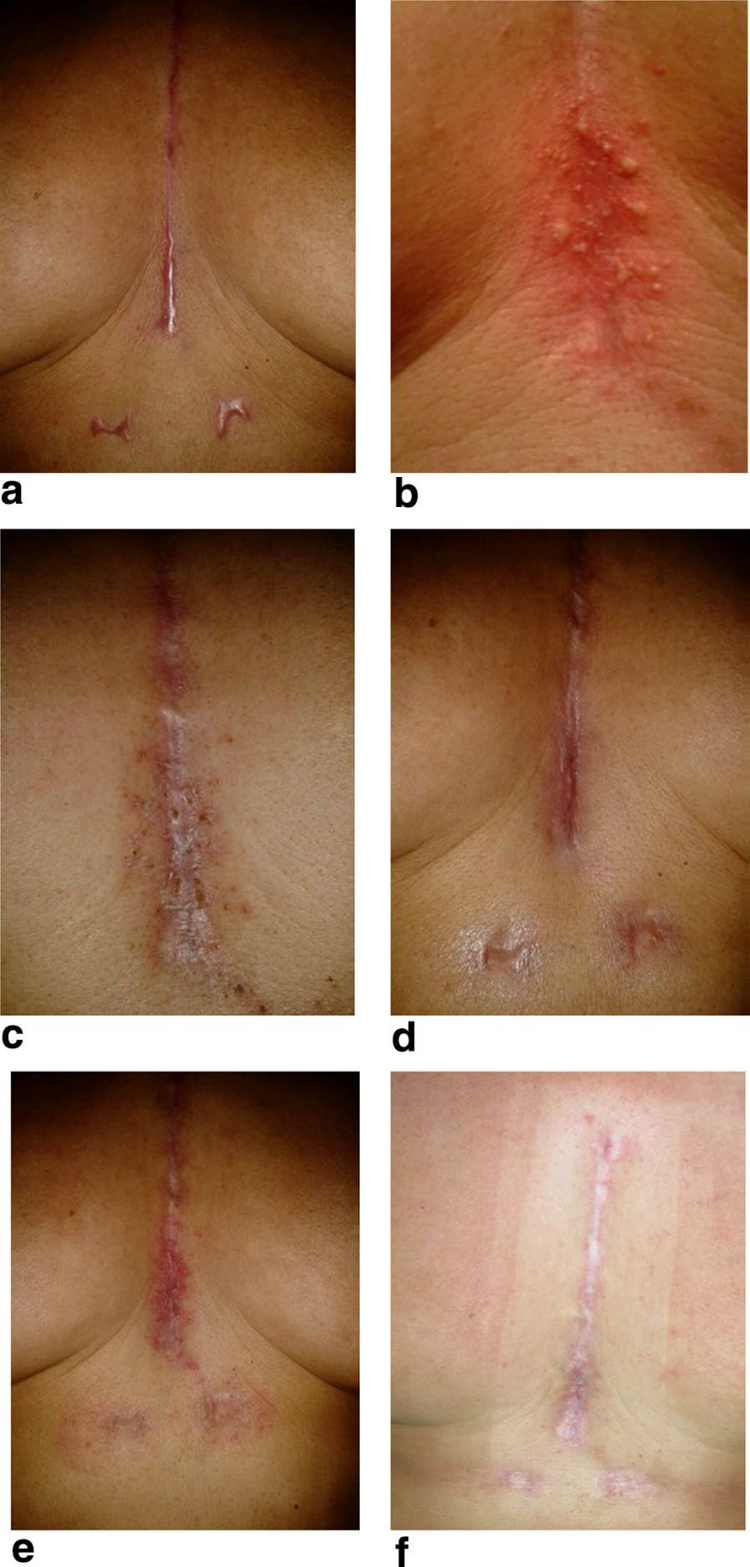
Patient‘s examination at 0 (**a**), 10 (**b**), 15 (**c**), 20 (**d**) 30 (**e**) and 50 (**f**) days after the ingenol-mebutate application performed on the lesion area

Here, ingenol mebutate gel was applied the first and the second day. The patient was examined at o, 10, 15, 20, 30 and 50 days after the application performed on the lesion area (Fig. 1a–f). On the day 10 of the application, a moderately crusting and oozing reaction was observed. According to the severity scale of local skin responses, which are the most common adverse effects of ingenol mebutate therapy [11], the composite score was 14/24: erythema: 4, flaking or scaling: 2, crusting: 4, swelling: 3, vesiculation or postulation: 1, erosion or ulceration: 0. In contrast, before treatment, the composite score was 12/24: erythema: 6, flaking or scaling: 0, crusting: 0, swelling: 6, vesiculation or postulation: 0, erosion or ulceration: 0. One month later, there was a clinical resolution of lesions, with a slightly squamous, post-inflammatory erythema (Fig. 1f).

## Real-time assay

**Fig. 2:**
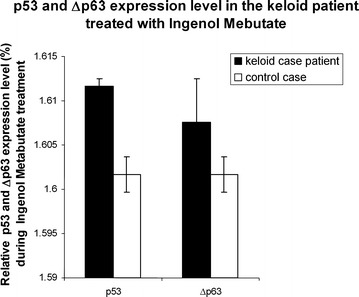
p53 and Δp63 expression level from a cutaneous biopsy, in the keloid case patient one month after treatment with ingenol-mebutate and in a control case

A second cutaneous biopsy, 1 month after treatment, proved the absence of residual keloids molecular markers. Real time assay was performed on the biopsy after treatment showing that ΔΝp63 and p53 was expressed almost as well as in hypertrophic scar and normal fibroblasts (Fig. 2). Control patient was a sex and age matched healthy patient. Usually, in keloid fibroblasts, ΔNp63 is overexpressed while p53 is under-expressed compared to normal fibroblasts [8].

All results shown are mean ± SD of at least three separate experiments, measuring each parameter by triplicate (n = 3). Statistical significant differences were tested by one way analysis of variance (ANOVA), and, when the *F* value was significant, by Student-Newman-Keul’s test. *P* value less than 0.05 (*) was considered statistically significant.

## Follow-up

The local tolerance of the treatment was acceptable for the patient and no systemic signs were observed. After 6 months of follow-up, the patient was still free from recidivant keloid scar.

The last follow-up of the patient, one year after treatment has not shown any clinical element that would suggest a flare up of the keloid disease. We have not taken a further skin biopsy for lack of clinical expression of keloid. At the present, the patient doesn’t display any keloid scar.

Future follow-up will be mainly clinical, but we plan also to perform more biopsies to confirm the absence of keloid.

## Discussion

Keloids are a grave psychological and physical dermatological condition for patients.

Keloid scarring is a raised scar which forms by expanding beyond the boundaries of the original lesion [12]. The most important histological manifestation of keloids is the overgrowth of atypical fibroblasts with excessive amassing of extra-cellular matrix components, particularly collagen, proteoglycans fibronectin and elastin, [13]. Keloid scar formation, differing from normal wound healing, starts with abnormal tissue growth in the dermal lesion extending beyond the borders of the original wound [13]. Suppression of apoptosis contributes to keloid development by means of accumulation of continuously proliferating cells, which account for the progressive and hypertrophic nature of keloids.

Despite the several studies performed on molecular mechanisms and treatment of keloids, the exact pathogenesis of such disease remains unknown and this makes therapies more problematic.

Current common treatments for keloids are often a combination of excision followed by a reconstructive surgical procedure [14]. Glucocorticoids or 5-fluorouracil injections followed by compression therapy such as silicone sheets are frequently employed. Nonetheless, recurrence remains between 45 and 100 %. Therefore, treatment of keloids is still a challenge. Surgery is the gold standard to treat any size of keloid, as it reduces the mass of the lesion. More strategies to prevent post-excision recurrence of keloids have been mentioned including silicone occlusive dressing, mechanical compression, radiation, cryosurgery, topical imiquimod application, bleomycin, intra-lesional injections of steroids, 5-fluorouracil as well as interferon alpha, -beta and -gamma [15].

In general, there is adequate evidence that several therapies have a synergistic effect when used together. Even if with limitations, significant improvement is possible with the available treatments. However, management of keloids remains difficult for high recurrence rates.

Lately, pro-apoptotic effects of ingenol-mebutate have been demonstrated [3]. Due to such effects, topical application of ingenol mebutate was revealed as being effective in humans for the treatment of precancerous skin; therefore we attempted to treat with ingenol mebutate the lesions from a patient showing recurrent keloids.

Our case report suggests that in some selected patients with keloids, ingenol mebutate could be considered as an alternative treatment. It could present the advantage, over usual treatment, that does not display potential recurrence or side effects, indeed, cutaneous adverse effects are shorter in duration with ingenol mebutate. The local tolerance of the treatment was acceptable for the patient, which after 1 year is still free of keloids. Additionally, the cutaneous biopsy proved the absence of residual keloids molecular markers as ΔΝp63 and p53.

## Conclusion

Ingenol mebutate could be considered as an alternative treatment option for keloids in patients, after other therapies have failed. Future studies and large number of samples are warranted to assess if ingenol mebutate is a suitable treatment for patients with keloids and to clarify the molecular mechanisms underlying the positive outcome of such treatment.

## Additional files

Additional file 1: Care check list.

## Abbreviations

p53 protein: Tumor suppressor p53
ΔNp63 protein: Isoform of the p53 homologue p63, which lacks an amino-terminal transactivation domain and antagonizes the induction of the gene expression by Deltap63
PKC-δ protein: Protein kinase C-delta
ATP: Adenosine triphosphate, coenzyme used as an energy carrier in the cells of all known organisms
CD8 (+) T cells: T cell with CD8 receptor that recognizes antigens on the surface of a virus-infected cell and binds to the infected cell and kill it
TNF-α: Tumor necrosis factor-alpha
PKC: Protein kinase C
MTT assays: Colorimetric assay for assessing cell metabolic activity. NAD(P)H-dependent cellular oxidoreductase enzymes may, under defined conditions, reflect the number of viable cells present. These enzymes are capable of reducing the tetrazolium dye MTT 3-(4,5-dimethylthiazol-2-yl)-2,5-diphenyltetrazolium bromide to its insoluble formazan, which has a purple color
