# Supplementary material for: Ingenol mebutate treatment in keloids
Source: BMC Res Notes. 2015 Sep 22;8:466. doi: 10.1186/s13104-015-1429-9 (PMC4578559; doi:10.1186/s13104-015-1429-9)
Supplement: Supplementary file 1 — Additional file 1: Care check list. [file 13104_2015_1429_MOESM1_ESM.docx]

** CARE Checklist (2013) of information to include when writing a case report**

| **Topic** | **Item** | **Checklist item description** | **Reported on Page** |
| --- | --- | --- | --- |
| **Title** | **1** | The words “case report” should be in the title along with the area of focus **Ingenol Mebutate in keloid: a case rep**  **report**  . . . .  . . . . . . . . . . . . . . . . . . . . . . . . | **1** |
| **Key Words** | **2**  **2** | 2 to 5 key words that identify areas covered in this case report. . . keloids, p53. Dp63, ingenol- mebutate . . . . . . . . . . . . . . . . . . . . . . . . . . . . . . . . . . . . . . . . . . . . . . . . . . . . | **2** |
| **Abstract** | **3a** | Introduction—What is unique about this case? What does it add to the medical literature? Keloid on the surgical scars, who has been treated , for the first time, with ingenol mebutate. . . . . . . . . . . . . . . . . . . . | **4** |
|  | **3b** | The main symptoms of the patient and the important clinical findings . . A 55-year-old woman presented with biopsy proven recurrent keloids on a presternal scar, subsequent to intervention of sternotomy, ran from nanubrrio to xiphoid appendix. The patient refused further workup for staging and was followed in the dermatology department. One year later, she presented recurrent keloids. Again, wide surgery was performed. Further physical examination was unremarkable. She presented with recurrent keloids adjacent to the surgical area | **5** |
|  | **3c** | The main diagnoses, therapeutics interventions, and outcomes . Since the patient refused categorically any further surgical intervention and the authors decided to attempt to treat these lesions with ingenol mebutate gel 0.015%, based on in vitro data on keloids cell lines [6-9]. Ingenol mebutate gel was applied the first and the second day. The patients was examinated at o, 10, 15, 20, 30 and 50 days after the application performed on the lesion area (Fig.1A-F).. On the day 10 of the application, a moderately crusting and oozing reaction was observed. According to the severity scale assessing ingenol mebutate toxicity, the composite score was  14/24: erythema: 4, flaking or scaling: 2, crusting: 4, swelling: 3, vesiculation or postulation: 1, erosion or ulceration: 0. One month later, there was a clinical resolution of lesions, with a slightly squamous, post-inflammatory erythema (Fig..1F).  . | **6** |
|  | **3d** | Conclusion—What are the main “take-away” lessons from this case? . Ingenol mebutate could be considered as an alternative treatment option for keloids in patients, after other therapies  have failed. Future studies and large number of samples are warranted to assess if ingenol mebutate is a suitable treatment for patients with keloids and to clarify the molecular mechanisms underlying the positive outcome of such treatment,  . . . . . . . . . . . . . . . . . . . . . . . . . . . . . . . . . . . . | **3** |
| **Introduction** | **4** | One or two paragraphs summarizing why this case is unique with references . No published data are available on a potential effect of ingenol mebutate on keloids (5).  . 5. [Cozzi SJ](http://www.ncbi.nlm.nih.gov/pubmed?term=Cozzi%20SJ%5BAuthor%5D&cauthor=true&cauthor_uid=22189786), [Ogbourne SM](http://www.ncbi.nlm.nih.gov/pubmed?term=Ogbourne%20SM%5BAuthor%5D&cauthor=true&cauthor_uid=22189786), [James C](http://www.ncbi.nlm.nih.gov/pubmed?term=James%20C%5BAuthor%5D&cauthor=true&cauthor_uid=22189786), [Rebel HG](http://www.ncbi.nlm.nih.gov/pubmed?term=Rebel%20HG%5BAuthor%5D&cauthor=true&cauthor_uid=22189786), [de Gruijl FR](http://www.ncbi.nlm.nih.gov/pubmed?term=de%20Gruijl%20FR%5BAuthor%5D&cauthor=true&cauthor_uid=22189786), [Ferguson B](http://www.ncbi.nlm.nih.gov/pubmed?term=Ferguson%20B%5BAuthor%5D&cauthor=true&cauthor_uid=22189786), [Gardner J](http://www.ncbi.nlm.nih.gov/pubmed?term=Gardner%20J%5BAuthor%5D&cauthor=true&cauthor_uid=22189786), [Lee TT](http://www.ncbi.nlm.nih.gov/pubmed?term=Lee%20TT%5BAuthor%5D&cauthor=true&cauthor_uid=22189786), [Larcher T](http://www.ncbi.nlm.nih.gov/pubmed?term=Larcher%20T%5BAuthor%5D&cauthor=true&cauthor_uid=22189786), [Suhrbier A](http://www.ncbi.nlm.nih.gov/pubmed?term=Suhrbier%20A%5BAuthor%5D&cauthor=true&cauthor_uid=22189786). Ingenol mebutate field-directed treatment of UVB-damaged skin reduces lesion formation and removes mutant p53 patches. J Invest Dermatol. 2012 Apr;132(4):1263-71.  . . . . . . . . . . . . . . . . . . . . | **4** |
| **Patient Information** | **5a** | De-identified demographic information and other patient specific information . . The patient lives in Campania, southern Italy, 55-year-old Caucasian woman presented with biopsy proven recurrent keloids on a presternal scar, subsequent to intervention of sternotomy, ran from nanubrrio to xiphoid appendix.  The patient refused further workup for staging and was followed in the dermatology department. One year later, she presented recurrent keloids. Again, wide surgery was performed. Further physical examination was unremarkable. She presented with recurrent keloids adjacent to the surgical area. . . . . . . . . . . . . . . . . . . . . . | **5** |
|  | **5b** | Main concerns and symptoms of the patient . .Recurrent keloid and .cosmetically unacceptable effects . . . . . . . . . . . . . . . . . . . . . . . . . . . . . . . . . . . . . . . . . . . . . . . . . . . |  |
|  | **5c** | Medical, family, and psychosocial history including relevant genetic information (also see timeline).  No family history of keloid. . | **5** |
|  | **5d** | Relevant past interventions and their outcomes . Wide surgery was performed. .but , she presented recurrent keloids . . . . . . . . . . . . . . . . . . . . . . . . . . . . . . . . . . . . . . . . . . . . . . . . | **2** |
| **Clinical Findings** | **6** | Describe the relevant physical examination (PE) and other significant clinical findings. . . The woman presented with biopsy proven recurrent keloids on a presternal scar.The patient presented recurrent keloids adjacent to the surgical area and she refused further surgery . . . | **4** |
| **Timeline** | **7** | Important information from the patient’s history organized as a timeline . . Recurrent keloids on a presternal scar, subsequent to intervention of sternotomy, ran from nanubrrio to xiphoid appendix.. Surgery,. one year later, she presented recurrent keloids. Again, wide surgery was performed. She presented keloids. again. . . . . . . . . . . . . . . . . . . . | **4** |
| **Diagnostic Assessment** | **8a** | Diagnostic methods (such as PE, laboratory testing, imaging, surveys). . . The diagnosis of a keloid was rather smooth and consisted in simple examination of the lesion and histologic examination. . . . . . . . . . . . . . . . . . . . . . . . . . . . | **5** |
|  | **8b** | Diagnostic challenges (such as access, financial, or cultural) . . The local tolerance of the treatment was acceptable for the patient and no systemic signs were observed . . . . . . . . . . . . . . . . . . . . . . . . . . . . . . . . | **4** |
|  | **8c** | Diagnostic reasoning including other diagnoses considered . . The diagnosis of a keloid was rather smooth and consisted in simple examination of the lesion and histologic examination . .. . . . . . . . . . . . . . . . . . . . . . . . . . . . . . . . . . | **4** |
|  | **8d** | Prognostic characteristics (such as staging in oncology) where applicable . . . . . . . . . . . . . . . . . . . . . . . . . . |  |
| **Therapeutic Intervention** | **9a** | Types of intervention (such as pharmacologic, surgical, preventive, self-care) . Since the patient refused categorically any further surgical intervention the authors decided to attempt to treat these lesions with ingenol mebutate based on in vitro data on keloids cell lines [6-9]. . . . . . . . . . . . . . . . . . . . . . . . . . . . . . . | **4** |
|  | **9b** | Administration of intervention (such as dosage, strength, duration) . Ingenol mebutate gel 0.015%, was applied the first and the second day.. . . . . . . . . . . . . . . . . . . . . . . .  . . . . . . . . . . . . . . . |  |
|  | **9c** | Changes in intervention (with rationale) . . . . . . . . . . . . . . . . . . . . . . . . . . . . . . . . . . . . . . . . . . . . . . . . . . . . . . . . . . . . . . . | **5** |
| **Follow-up and**  **Outcomes** | **10a** | Clinician and patient-assessed outcomes (when appropriate) .. After 6 months of follow-up the patient was still free of keloids recurrence . . . . . . . . . . . . . . .. . . . . . . . . . . . . . . . . . . . . . . . . . . . | **5** |
|  | **10b** | Important follow-up diagnostic and other test results . . . One month later, there was a clinical resolution of lesions, with a slightly squamous, post-inflammatory erythema. . . . . .. . . . . . . . . . . . . . . . . . . . . . . . . . . . . . . . . . . . . . . . . . . . . . | **5** |
|  | **10c** | Intervention adherence and tolerability (How was this assessed?) . . The local tolerance of the treatment was acceptable for the patient and no systemic signs were observed . . . . . . . . . . . . . . . . . . . . . . . . . . . . . . . . . . . . . . . | **5** |
|  | **10d** | Adverse and unanticipated event. On the day 10 of the application, a moderately crusting and oozing reaction was observed. According to the severity scale assessing ingenol mebutate toxicity, the composite score was 14/24: erythema: 4, flaking or scaling: 2, crusting: 4, swelling: 3, vesiculation or postulation: 1, erosion or ulceration: 0. One month later, there was a clinical resolution of lesions, with a slightly squamous, post-inflammatory erythema. . . . . . . . . . . . . . . . . . . . . . . . . . . . . . . . . . . . . . . . . . . . . . . . . . . . . . . . . . . | **4** |
| **Discussion** | **11a** | Discussion of the strengths and limitations in your approach to this case . . . . Some selected patients with keloids, ingenol mebutate could be considered as an alternative treatment. It could present the advantage over usual treatment not to display potential recurrence or side effects, indeed, cutaneous adverse effects are shorter in duration with ingenol mebutate. The local tolerance of the treatment was acceptable for the patient, which after six months is still free of keloids. Beside, the cutaneous biopsy proved the absence of residual keloids molecular markers as Δp63 and p53. | **6** |
|  | **11b** | Discussion of the relevant medical literature. . . . Lately, pro-apoptotic effects of ingenol- mebutate have been demonstrated (3). Topical application of ingenol mebutate was revealed as being effective in humans for the treatment of precancerous skin.. Topical application of ingenol mebutate was revealed as being effective for human and murine melanoma in mouse models (4).. Ingenol mebutate treatment also reduced the number of mutant p53 keratinocyte patches by about 70% . No published data are available on a potential effect of ingenol mebutate on keloids (5).  . . . . . . . . . . . . . . . . . . . . . . . . . . . . . . . . . . . . . . . . . . . . . . . . . . | **6, 3** |
|  | **11c** | The rationale for conclusions (including assessment of possible causes) .  Future studies and large number of samples are warranted to assess if ingenol mebutate is a suitable treatment for patients with keloids and to clarify the molecular mechanisms underlying the positive outcome of such treatment . . . . . . . . . . . . . . . . . . . . . . . . . . . . . . . . | **6** |
|  | **11d** | The primary “take-away” lessons of this case report . . . Ingenol mebutate could be considered as an alternative treatment option for keloids in patients, after other therapies  have failed. . . . . . . . . . . . . . . . . . . . . . . .. . . . . . . . . . . . . . . . . . . . . . . . | **6** |
| **Patient Perspective** | **12** | When appropriate the patient should share their perspective on the treatments they received . . . . . . YES. . . . . . . . . . . |  |
| **Informed Consent** | **13** | Did the patient give informed consent? Please provide if requested . . . . . . . . . . . . . . . . . . . . . . . . . . . . . . . . . . . . . . | **Yes x** **No** |
